# TRIM21 regulation of IRF-mediated type I interferon signaling in systemic autoimmune diseases

**DOI:** 10.3389/fimmu.2025.1695818

**Published:** 2025-11-26

**Authors:** Kailai Panlu, Yihan Wang, Xinchao Zhu, Zhiting Lin, Ting Fang, Xinyi Yao, Zhimin Xie, Xinchang Wang

**Affiliations:** 1 The Second School of Clinical Medicine, Zhejiang Chinese Medical University, Hangzhou, China; 2 Department of Rheumatology, The Second Affiliated Hospital of Zhejiang Chinese Medical University, Hangzhou, China

**Keywords:** TRIM21, type I interferon, IRF family, autoimmune diseases, ubiquitination

## Abstract

Dysregulation of type I interferon (IFN-I) signaling has been implicated in the pathogenesis of multiple systemic autoimmune diseases, including systemic lupus erythematosus, Sjögren’s syndrome, and rheumatoid arthritis. Tripartite motif-containing protein 21 (TRIM21) serves as both a major autoantigen and a pivotal E3 ubiquitin ligase. In the IFN-I pathway, TRIM21 plays a dual regulatory role by targeting transcription factors such as interferon regulatory factor 3 (IRF3), interferon regulatory factor 5 (IRF5), and interferon regulatory factor 7 (IRF7) through multiple ubiquitination mechanisms. This duality enables TRIM21 to both activate IFN-I signaling and mediate its negative feedback, thus maintaining immune homeostasis. However, the presence of anti-TRIM21 autoantibodies may impair its ubiquitin ligase function, resulting in persistent activation of the IFN-I pathway and chronic inflammation. This review summarizes the mechanisms by which TRIM21 regulates IRF family members across various tissues and immune contexts, and explores how its dysfunction contributes to tissue-specific inflammatory responses. Furthermore, we evaluate the potential diagnostic and stratification value of anti-Ro52 antibodies and propose TRIM21 as a novel upstream therapeutic target to restore interferon balance in autoimmune diseases.

## 1 Introduction

Autoimmune diseases arise from dysregulated immune homeostasis, leading to exaggerated host immune responses (1–3). Although their etiology is complex and multifactorial, aberrant activation of the type I interferon (IFN-I) signaling pathway is widely recognized as a shared mechanism underlying the development of diverse autoimmune disorders (4, 5). Sustained overproduction of IFN-I drives chronic inflammation and progressive tissue damage (6–8). Consequently, elucidating the key molecules and regulatory mechanisms that maintain IFN-I homeostasis is essential both for understanding disease pathogenesis and for informing the development of targeted therapeutic strategies.

The tripartite motif-containing (TRIM) protein family is a class of E3 ubiquitin ligases characterized by conserved domains, including a RING finger, B-box, and coiled-coil region. These proteins are broadly involved in biological processes such as cell cycle regulation, autophagy, and innate immune responses (9). An important member of this family, Tripartite motif-containing protein 21 (TRIM21), also known as Ro52, functions as an E3 ubiquitin ligase and is a common autoantigen. Interferon regulatory factor (IRF) proteins are its principal substrates for ubiquitination-mediated regulation (10, 11). Through distinct ubiquitination mechanisms, TRIM21 can either activate or degrade key proteins in the interferon pathway, such as interferon regulatory factor 3 (IRF3), interferon regulatory factor 5 (IRF5), and interferon regulatory factor 7 (IRF7). In this way, it exerts bidirectional control over IFN-I signaling by promoting activation under specific conditions and mediating negative feedback regulation to limit excessive responses. However, the context dependence of these functions remains poorly understood, as it is influenced by a range of factors including cell type, stimulation pattern, target protein, and the type of ubiquitin chain involved. Such a gap limits the translation of current mechanistic insights into a deeper understanding of disease pathogenesis and the development of targeted interventions.

Interestingly, the enzymatic activity of TRIM21 is itself regulated by its ubiquitination status (12). However, this critical self-regulatory phenomenon and its role under pathological conditions have not yet been systematically elucidated, which limits our comprehensive understanding of its immune-regulatory functions and its potential as a therapeutic target.

This review systematically elucidates the dual mechanisms by which TRIM21 regulates the IFN-I signaling pathway. Particular attention is given to how its E3 ubiquitin ligase activity is activated or suppressed through different types of ubiquitin chains. We also discuss the relevance of these processes to the disruption of immune homeostasis observed in autoimmune diseases. Ultimately, we evaluate the feasibility of targeting TRIM21 to modulate IFN-I homeostasis and explore its prospects for clinical translation, offering new perspectives for the precision treatment of autoimmune diseases.

## 2 Structure and molecular mechanism of TRIM21 protein

### 2.1 Structural domain composition of TRIM21 protein

**Figure 1:**
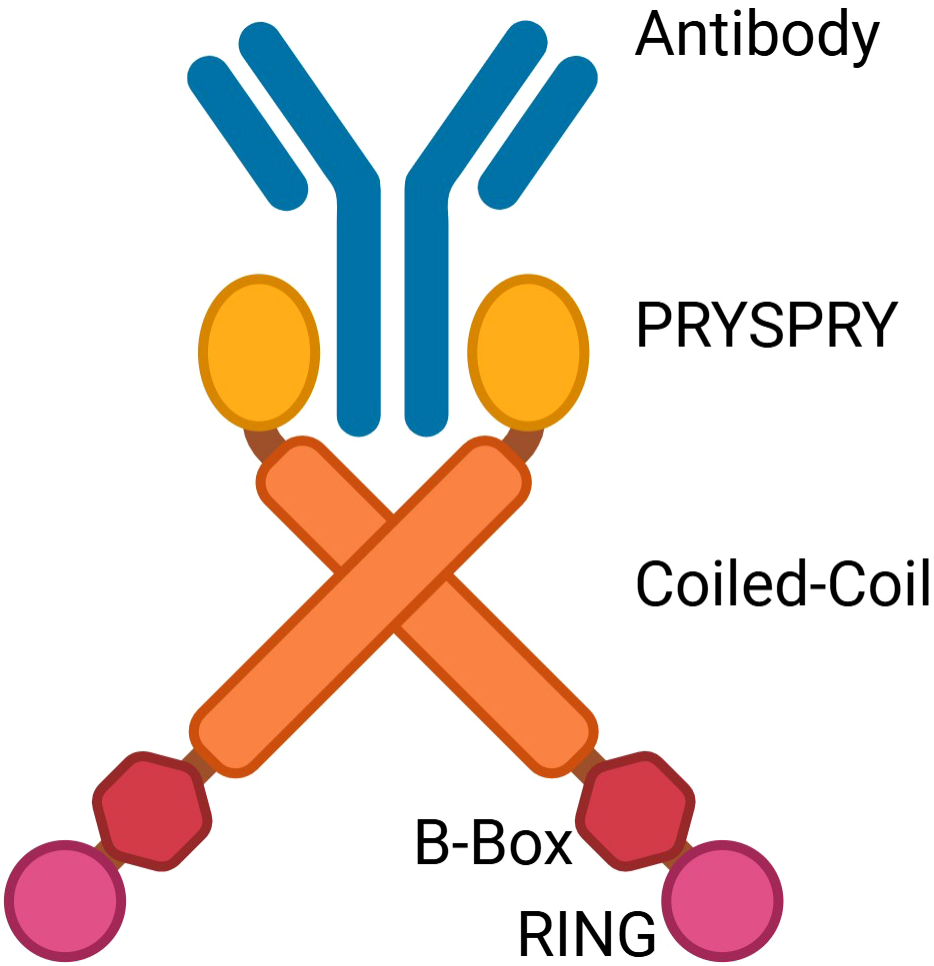
Illustration showing binding of dimeric full-length TRIM21 to antibody (blue). The RING (pink), B-Box (red), Coiled-Coil (orange), and PRYSPRY (yellow) domains of TRIM21 are shown. Image 1 was made in BioRender™.

TRIM21 is a cytosolic antibody receptor and E3 ubiquitin ligase (13). It plays a central role in the regulation of interferon signaling and innate immune responses. From the N-terminus to the C-terminus, TRIM21 contains four conserved domains: RING, B-box, coiled-coil (CC), and PRY/SPRY (14) (**Figure 1**).

These domains collectively confer TRIM21’s unique functions. The RING domain is characterized by a series of conserved cysteine and histidine residues (15). It provides E3 ubiquitin ligase activity and mediates the formation of multiple types of polyubiquitin chains, including K48-linked chains that target proteins for degradation and K63-linked chains that regulate signal transduction (16–18). TRIM21 becomes activated after monoubiquitination at its N-terminus (RING domain) by the E2 enzyme Ube2W. This priming modification facilitates the recruitment of the Ube2N/Ube2V2 heterodimer, initiating the K63-specific ubiquitination process (19).

TRIM21’s B-box domain coordinates a Zn²^+^ ion. This metal coordination is essential for maintaining its tertiary structure and molecular stability. By modulating the spatial distance between adjacent domains, this interaction may indirectly influence the E3 ligase activity of TRIM21 (12). The B-box domain exerts an autoinhibitory role by modulating RING activity. It can mimic the E2 ubiquitin-conjugating enzyme and occupy the catalytic site of the RING domain. In this autoinhibitory state, the ubiquitin ligase activity is suppressed, thereby preventing excessive pro-inflammatory signaling (11, 20–22). Notably, overexpression of the kinases IKKβ or TBK1 induces phosphorylation at the LxxIS motif within the RING domain. This phosphorylation event relieves B-box–mediated inhibition (20). These findings may be of particular interest in autoimmune contexts, as disruption of this autoinhibitory mechanism could lead to excessive pro-inflammatory signaling.

The coiled-coil (CC) domain forms a superhelical bundle of α-helices. It is essential for the homotypic assembly of TRIM family proteins and for the self-association of TRIM21 (18, 22). Studies have shown that dimerization of the catalytic RING domain is essential for the ubiquitination activity of TRIM21 (23). This homotypic interaction promotes TRIM21 homodimerization, thereby enhancing the E3 ubiquitin ligase activity of the RING domain (23–25). The PRY/SPRY domain contains a high-affinity Fc-binding site, which enables TRIM21 to bind various immunoglobulins (26). Notably, the mode of Fc recognition by the PRY/SPRY domain of TRIM21 differs markedly from that of classical Fc receptors such as FcγR and C1q (26–29). It can bind to multiple antibody isotypes, including immunoglobulin G (IgG), immunoglobulin M (IgM), and immunoglobulin A (IgA) (30, 31). The strong Fc-binding affinity of this domain may contribute to epitope exposure or the formation of antibody-targeted complexes. However, conclusive evidence supporting this function is currently lacking.

### 2.2 Self-regulated ubiquitination mechanism of TRIM21: special E3 ligase activity control mode

**Figure 2:**
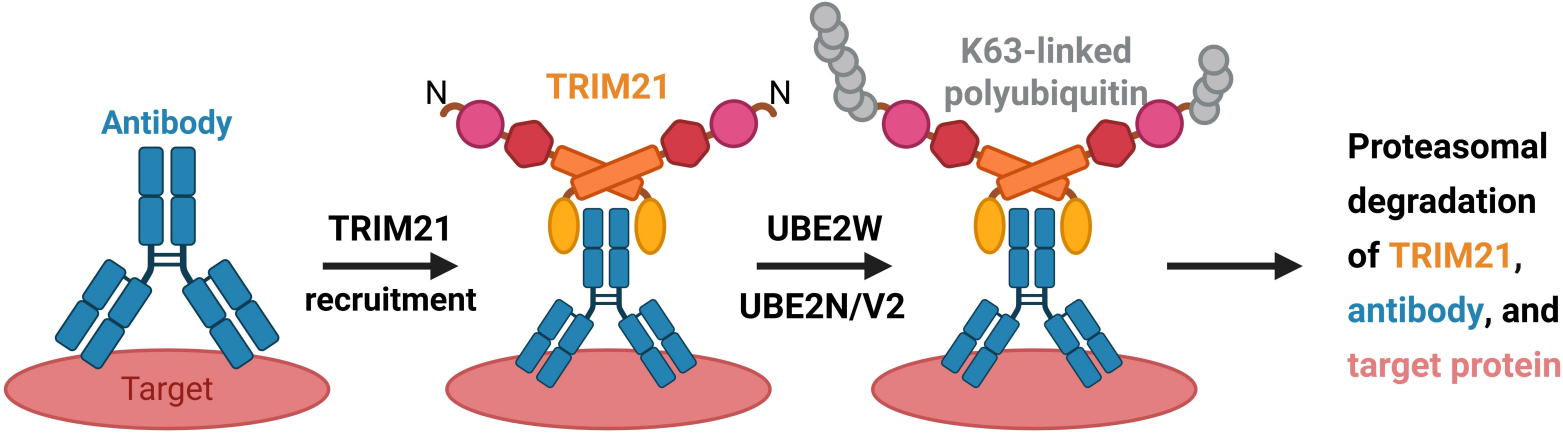
Simplified schematic of the current Trim-Away model. The target protein (pink) is bound by an antibody (blue), which in turn is recognized by TRIM21 (yellow). Subsequent TRIM21 activation results in sequential monoubiquitination and polyubiquitination of its N terminus with K63-linked polyubiquitin, which requires the E2 ubiquitin-conjugating enzymes UBE2W and UBE2N/UBE2V2, respectively. The polyubiquitin chain on TRIM21 has been proposed to promote the proteasomal degradation of all three components. Image 2 was made in BioRender™.

Self-ubiquitination is a key mechanism by which E3 ligases fine-tune their enzymatic activity and functional state. For TRIM21, this mechanism is essential for the functional activation of its E3 ligase activity (12). Under physiological conditions, the E3 ligase activity of TRIM21 is autoinhibited by its B-box domain and is activated only upon recognition of specific targets, such as antibody-coated adenoviruses, thereby triggering immune signal transduction and target protein degradation (32, 33) (**Figure 2**).

The activation of TRIM21 is orchestrated through multiple coordinated steps. Using K63-linked ubiquitination as an example, TRIM21 engages two distinct E2 enzymes upon dimerization (34, 35). Initially, UBE2W catalyzes monoubiquitination at the N-terminus of TRIM21 (35). This is followed by the recruitment of UBE2N/UBE2V2, which extends the modification by assembling a K63-linked polyubiquitin chain from the monoubiquitin (36). Due to the directional specificity of UBE2N/UBE2V2, the TRIM21 dimer is unable to elongate its own ubiquitin chain in cis and instead relies on a trans mechanism (37). This process involves the juxtaposition of at least three TRIM21 RING domains. Two of them assemble into a dimer that acts as the catalytic core, responsible for recruiting and activating the UBE2N~Ub conjugate. The activated dimer subsequently transfers ubiquitin to a third TRIM21 molecule that has been monoubiquitinated, thereby promoting chain elongation (19). This substrate is positioned approximately 9 nm from the RING dimer. Through the trans mechanism, its chain is further elongated from a monoubiquitin to a tetraubiquitin. Once the chain reaches a sufficient length, the K63-linked ubiquitin chain on the original RING dimer can subsequently be extended in cis, thereby enhancing the dimer’s ability to ubiquitinate additional target proteins (19).

In summary, functional activation of TRIM21’s E3 ubiquitin ligase activity depends on its self-ubiquitination. Formation of a functional self-ubiquitin chain requires sequential steps: relieving B-box-mediated autoinhibition, promoting RING domain dimerization, and establishing a specific RING topology—events that are triggered only upon substrate binding (11, 12, 19). This unique self-regulatory mechanism ensures that TRIM21’s ubiquitination activity is initiated exclusively in response to pathogen recognition or abnormal complexes, thereby preventing excessive inflammatory responses and potential autoimmune damage.

In conventional protein degradation pathways, E3 ligase-mediated ubiquitination typically results in degradation of the substrate alone (38). In contrast, TRIM21 produces its own ubiquitin chain upon substrate binding. This not only targets the bound substrate for degradation but also facilitates the degradation of the antibody and TRIM21 itself. This built-in mechanism effectively limits prolonged inflammatory signaling. However, when this regulatory mechanism becomes dysregulated, aberrant activation may contribute to the development of autoimmune diseases (31, 33).

### 2.3 TRIM21 functions through specific ubiquitination

TRIM21 initiates ubiquitination only after binding to specific target molecules, which ensures the precision of its signaling regulation (32, 33). Simultaneously, TRIM21 activates multiple signaling pathways, including the nuclear factor kappa-light-chain-enhancer of activated B cells (NF-κB) and activator protein 1 (AP-1) pathways, and promotes the activation of transcription factors such as IRF3, IRF5, and IRF7. These events lead to the production of pro-inflammatory cytokines and the initiation of innate immune responses. Concurrently, TRIM21 activates multiple signaling pathways, including the NF-κB and AP-1 pathways, and promotes the activation of transcription factors such as IRF3, IRF5, and IRF7 (39). These events lead to the production of pro-inflammatory cytokines and the initiation of innate immune responses (35, 39).

These functions are closely linked to TRIM21’s capacity to assemble various types of ubiquitin chains. During ubiquitination, the E3 ligase determines the substrate to be modified, whereas the E2 enzyme specifies the type of ubiquitin linkage that will be formed (40). TRIM21 can catalyze the formation of distinct ubiquitin chain types by cooperating with specific E2 enzymes, thereby exerting diverse biological functions (41–43). In particular, K48- and K63-linked polyubiquitin chains have been investigated most extensively (44). The RING dimer of TRIM21 can undergo auto-monoubiquitination via E2 enzymes such as UbcH6, Ube2E2, UbcM2, or Ube2W. This monoubiquitin can subsequently be extended by Ube2N/Ube2V2 or Ube2K into K63- or K48-linked polyubiquitin chains, respectively (45). Among these, the K48-linked chains primarily target proteins for proteasomal degradation, whereas K63-linked chains are mainly involved in signaling processes (19, 44).

The functional role of K63-linked ubiquitin chains remains a subject of ongoing debate. In the past, K63-linked chains have been generally viewed as primarily involved in signal transduction and not associated with proteasomal degradation pathways (46–48). But some studies have indicated that in the context of antibody-coated pathogens engaging TRIM21, K63-linked chains may contribute to both downstream signaling and target protein degradation (35, 49, 50). In addition, studies have shown that K63- and K48-linked chains can cooperate to form mixed ubiquitin chains (35). During the Trim-Away process, K48/K63 branched ubiquitin chains have been detected, suggesting that once K63 chains are established, K48 linkages may be incorporated in a branched configuration to coordinate the regulation of intracellular signaling and degradation pathways (37). The precise impact of these branched chains on protein degradation needs to be fully elucidated.

## 3 Bidirectional regulation of TRIM21 on type I interferon pathway

IRFs are the core transcription factors of the IFN-I signaling pathway. Numerous studies have demonstrated that the expression and activation of IRF family members, including IRF3, IRF5, and IRF7, are significantly increased in systemic autoimmune diseases such as systemic lupus erythematosus (SLE) and Sjögren’s syndrome (SS) (51, 52). Because their activity in peripheral blood closely reflects both IFN-I levels and disease activity, these IRFs are widely regarded as potential clinical biomarkers (53–55). Studies have confirmed that TRIM21 regulates the stability and function of multiple IRF family members through ubiquitination, suggesting that it may modulate IFN-I pathway activity and contribute to the pathogenesis of autoimmune diseases by altering IRF activity (56). However, the precise nature of this regulation, specifically whether it leads to activation or degradation of IRFs under different cell types or immune contexts, remains controversial and requires further elucidation (56–60).

### 3.1 Negative regulation: TRIM21 suppresses excessive type I interferon signaling

TRIM21, a critical regulator of immune homeostasis, mediates the ubiquitination and subsequent proteasomal degradation of IRFs, thereby restraining excessive IFN-I signaling and preserving immune equilibrium (17, 56, 60). This feedback mechanism is vital for constraining aberrant immune activation (61). Its disruption may precipitate widespread autoimmune responses (62). For example, TRIM21-deficient mice exhibit uncontrolled systemic inflammation and an autoimmune phenotype reminiscent of SLE following minor tissue injury (63). Pro-inflammatory cytokines can be excessively produced without TRIM21-mediated negative regulation. This may represent a key molecular mechanism driving autoimmune responses and widespread inflammation (60, 64).

#### 3.1.1 IRF3

IRF3 is a pivotal transcription factor in the IFN-I signaling pathway (65). It regulates the expression of interferon genes such as interferon-beta (IFN-β), thereby initiating cellular antiviral responses (56). Upon activation of Toll-like receptor 3 (TLR3) or Toll-like receptor 4 (TLR4), TRIM21 mediates the polyubiquitination and subsequent proteasomal degradation of IRF3 (56). However, the type of ubiquitin chain involved remains controversial. For example, during Sendai virus infection, TRIM21 promotes IRF3 degradation via K48-linked ubiquitination. In contrast, during African swine fever virus infection, TRIM21 induces IRF3 degradation through K63-linked ubiquitination (66). This difference may reflect virus-specific mechanisms. African swine fever virus non-structural protein MGF360-14L has been shown to interact with IRF3 and mediate its degradation via K63-linked ubiquitin chains, thereby suppressing the IFN-I response (67).

#### 3.1.2 IRF7

IRF7 is a crucial regulator of the IFN-I response. It acts in coordination with IRF3 to initiate and amplify the production of interferon-alpha (IFN-α) and IFN-β (68). Deficiency of IRF7 markedly impairs the IFN-I response, particularly reducing IFN-α secretion by plasmacytoid dendritic cells (pDCs) (69–71). IRF7 plays an important role in the pathogenesis of autoimmune diseases such as SLE, and its upregulation is closely associated with disease susceptibility and activity (72–79).

After the IFN-I signaling pathway is activated, TRIM21 binds to IRF7, mediates its polyubiquitination, and promotes its degradation by the proteasome. The K48 and K63-linked ubiquitin chains participate in this process, where the K63 chain is formed by TRIM21 itself and promotes the K48 chain polyubiquitination of IRF7 (56, 80).

In addition, TRIM21 can indirectly regulate IRF7 by modulating the ubiquitination of N-Myc (and STAT) interactor (NMI), an interferon-inducible protein that acts upstream of IRF7. NMI promotes IRF7 degradation through the proteasome pathway (81, 82).

The SPRY domain of TRIM21 interacts with the coiled-coil domain of NMI and mediates K63-linked ubiquitination at lysine 22 of NMI (81), leading to NMI activation. This modification enhances both the formation and stability of the NMI/interferon-induced 35 kDa protein complex. Through this mechanism, TRIM21 indirectly promotes IRF7 degradation.

#### 3.1.3 IRF5

IRF5 is a key transcription factor that regulates B cell differentiation and promotes autoantibody production by self-reactive B cells (83, 84). IRF5 drives inflammatory responses by stimulating dendritic cells, macrophages, and monocytes to secrete pro-inflammatory cytokines such as IL-6, IL-12, IL-23, and TNF-α, thereby playing a pivotal role in immune responses (85–87). Additionally, IRF5 polymorphisms are strongly associated with SLE and SS (88–93). In various autoimmune diseases, including rheumatoid arthritis and SLE, excessive IRF5 activation is closely linked to dysregulated inflammatory responses (18, 94, 95).

Following TLR7- and TLR9-mediated activation of IRF5, TRIM21 regulates B cell function by ubiquitinating IRF5 and facilitating its degradation (83, 84). Overexpression of TRIM21 markedly reduces IRF5 expression in B cell lines, attenuates proliferative signaling in B cells, and promotes apoptosis (83). Conversely, TRIM21 dysfunction may promote abnormal B cell activation and proliferation, thereby contributing to the development of autoimmune diseases (96).

### 3.2 Positive regulation: TRIM21 promotes type I interferon signaling

In addition to negatively regulating IRF activity, TRIM21 functions as a positive modulator of immunity by ubiquitinating upstream signaling components, thereby activating IRFs and amplifying IFN-I signaling. During human adenovirus type 5 infection, TRIM21 catalyzes K63-linked ubiquitination to activate the IKKα–IKKβ–NEMO and TAK1–TAB1–TAB2 signaling complexes. This, in turn, activates NF-κB, AP-1, and the transcription factors IRF3, IRF5, and IRF7, thereby triggering IFN-I responses, promoting pro-inflammatory cytokine production, and establishing an antiviral state (32, 35).

Moreover, TRIM21 directly engages IRF3 and IRF7 to potentiate their transcriptional activity. The following sections detail the mechanisms through which TRIM21 positively regulates members of the IRF family.

#### 3.2.1 IRF3

TRIM21 can activate upstream regulators of IRF3. During coxsackievirus B3 infection, TRIM21 mediates K27-linked ubiquitination of mitochondrial antiviral signaling protein (MAVS) through an intramolecular interaction between its RING and PRY/SPRY domains, thereby promoting the recruitment of TBK1 to MAVS. This promotes IRF3 phosphorylation and dimerization, leading to the induction of IFN-I responses and strengthening host innate immunity against RNA viruses (97, 98).

TRIM21 also contributes to the stabilization of IRF3. Ectopic expression of TRIM21 enhances IRF3-driven gene transcription, whereas TRIM21 knockdown reduces this activity. The peptidyl-prolyl cis-trans isomerase NIMA-interacting 1 (Pin1) interacts with phosphorylated IRF3 and facilitates its degradation via the ubiquitin–proteasome pathway (57). TRIM21 directly binds to IRF3 via its Fc domain, interferes with Pin1-mediated conformational changes, blocks the recruitment of E3 ubiquitin ligases by IRF3, and delays its ubiquitin-mediated degradation (57). By inhibiting Pin1, TRIM21 indirectly stabilizes activated IRF3, maintains its homeostasis, and promotes downstream interferon signaling.

#### 3.2.2 IRF8

IRF8 is a key transcription factor involved in maintaining immune system homeostasis. Genetic polymorphisms of IRF8 have been linked to increased susceptibility to various immune-related diseases, while functional defects can result in severe immunodeficiency syndromes (99–102).

Studies have shown that TRIM21 directly interacts with IRF8 and mediates its ubiquitination. However, unlike conventional ubiquitination, TRIM21-mediated ubiquitination of IRF8 does not induce protein degradation. It upregulates IRF8-driven cytokine expression. In macrophages stimulated by interferon-γ or TLR ligands, TRIM21 binds to IRF8 and promotes its ubiquitination. However, this mechanism is not yet fully understood. This modification did not lead to IRF8 degradation but promoted IRF8-induced IL-12 expression. When the adaptor protein p62 formed a complex with TRIM21 and IRF8, IRF8 ubiquitination was further enhanced. Paradoxically, this modification led to downregulation of IL-12p40 expression (59).

### 3.3 TRIM21 maintains the dynamic balance of the type I interferon signaling through distinct modes of ubiquitination.

In response to IFN-I, TRIM21 expression is upregulated and it promotes the degradation of IRF3, IRF5, and IRF7 through K48-linked ubiquitination. This forms a negative feedback loop that limits excessive IFN-I production and helps prevent the onset of autoimmune diseases (11). Although the specific type of ubiquitin chain involved in IRF3 degradation remains controversial, some studies suggest that TRIM21 mediates this process through either K48- or K63-linked chains. Nevertheless, there is broad agreement that these modifications ultimately target IRF3 for proteasomal degradation, thereby dampening IFN-I signaling (18, 56, 103).

In addition to its negative regulatory role, TRIM21 also positively regulates IFN-I signaling by catalyzing K63- or K27-linked ubiquitination of upstream signaling molecules such as MAVS, the IKK complex, and TAK1. This leads to the activation of IRF3, IRF5, and IRF7, thereby promoting proinflammatory cytokine production (97, 98). Moreover, TRIM21 exerts a direct stabilizing effect on IRF3 and IRF8. These mechanisms reveal the multifaceted roles of TRIM21 in regulating IFN-I responses (57, 58).

In addition to regulating IRFs, TRIM21 also modulates other key components of the interferon signaling pathway through diverse ubiquitination mechanisms, exerting both activating and inhibitory effects on IFN-I responses. For example, TRIM21 mediates K27-conjugated polyubiquitination of MAVS and up-regulates IRF3-induced type I IFN (97, 98). In another mechanism,TRIM21 mediates K63-linked ubiquitination that interferes with the autophagic clearance of STING, leading to its stabilization and enhanced type I interferon responses (104). These findings indicate that TRIM21 can strengthen the antiviral response by facilitating the activation of upstream adaptor molecules in both RNA and DNA sensing pathways.

Conversely, TRIM21 can also function as a negative regulator of interferon signaling. It has been reported that TRIM21 promotes K48-linked ubiquitination and proteasomal degradation of the cytosolic DNA sensor DDX41, thereby suppressing DDX41-mediated IFN-I production as a feedback control mechanism (105). Similarly, IFI16, another important DNA sensor, was shown to undergo TRIM21-mediated ubiquitination and degradation, further contributing to the negative regulation of DNA-triggered interferon signaling (106). TRIM21 also directly binds to STING, promoting its ubiquitination and subsequent proteasomal degradation, although the specific ubiquitin linkage involved has not yet been determined (106).

**Figure 3:**
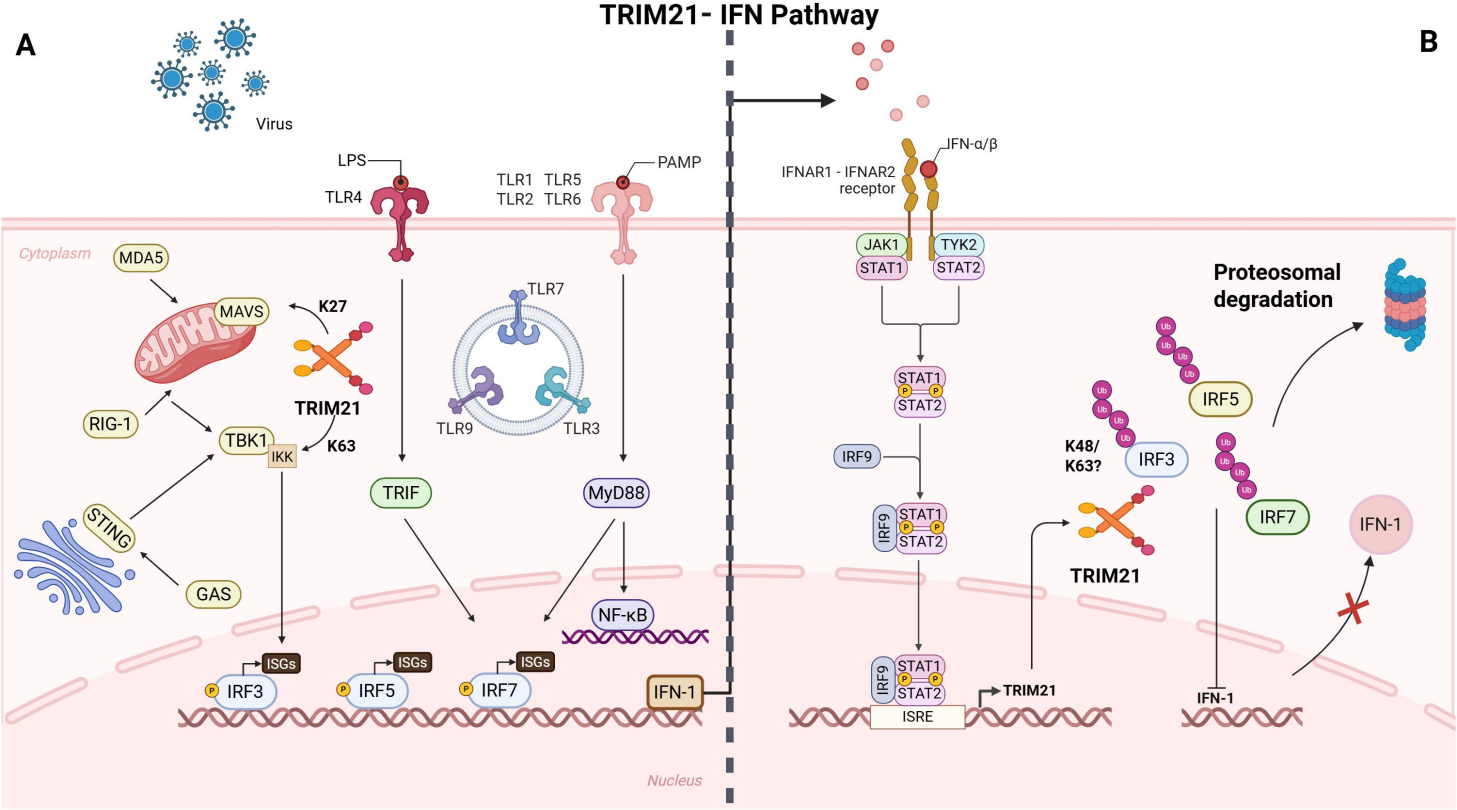
Different signaling pathways are involved in the inflammatory regulation of IFN-I. **(A)** After being stimulated by viruses, PAMP, etc. in the external environment, the DNA sensors activate STING, which moves to the Golgi and is phosphorylated by TBK1, allowing for the phosphorylation and activation of IRF 3. Upon binding to their ligands, RIG-I and MDA5 engage MAVS, leading to activation of TBK1 and members of the IKK family of kinases. Similarly, TLRs signal through MyD88 and TRIF adaptor molecules, leading to the activation of TBK1 and members of the IKK family. These kinases trigger the phosphorylation, activation, and dimerization of IRFs and the release of NF-κB. IRFs and NF-κB then migrate into the nucleus where they bind to promoter regions of IFN-I and other target genes, thereby stimulating IFN-I as well as anti-inflammatory and pro-inflammatory cytokine gene transcription. **(B)** Type I interferons (primarily IFN-α/β) bind to the cell surface receptor IFNAR2, triggering the formation of a functional receptor complex with IFNAR1. This interaction leads to the phosphorylation of JAK1 and TYK2, which in turn recruit and phosphorylate STAT proteins, promoting their dimerization, trimerization, and nuclear translocation. The ISGF3 complex, composed of STAT1, STAT2, and IRF9, recognizes the ISRE motif in the nucleus and induces the transcription of interferon-stimulated genes (ISGs) including TRIM21. Upregulated TRIM21 in turn downregulates IFN-I signaling by targeting IRF3, IRF5, and IRF7 for ubiquitination. Image 3 was made in BioRender™.

Taken together, these results position TRIM21 as a central coordinator of IFN-I signaling, bridging its canonical regulation of IRFs with broader control over upstream adaptor molecules to maintain immune equilibrium (**Figure 3**).

Overall, TRIM21 orchestrates a highly intricate regulation of the type I interferon (IFN-I) pathway. By assembling different types of ubiquitin chain linkages, it can either activate or degrade critical members of the IRF family, thereby maintaining immune homeostasis under diverse immunological conditions. This dual functionality not only advances our understanding of innate immune regulation but also provides novel insights into the pathogenesis of autoimmune diseases and potential avenues for therapeutic intervention. The following section will examine the pathological roles of IRFs in autoimmune diseases. It will focus on how TRIM21 dysfunction causes abnormal IRF activity, disrupts immune balance, and drives disease progression.

## 4 TRIM21 ubiquitination regulation imbalance and autoimmune diseases

### 4.1 The role of TRIM21 in regulating the type I interferon signaling in autoimmunity

The IFN-I system is critical for preserving immune homeostasis. Disruption of IFN-I regulation is considered a major trigger for autoimmune activation. Sustained overactivation of IFN-I signaling has been implicated as a central pathogenic mechanism in SLE, rheumatoid arthritis (RA), SS, and other autoimmune diseases (4, 107, 108). IRF3, IRF5, and IRF7 are central transcription factors in the regulation of type I interferon signaling. Genetic polymorphisms in these IRFs or in their upstream regulation by cytosolic nucleic acid sensors may disrupt IFN-I homeostasis and increase susceptibility to systemic autoimmune diseases (52).

TRIM21 is a major autoantigen in autoimmune diseases such as SS and SLE (10, 109, 110). In recent years, it has gained increasing attention as a pivotal E3 ubiquitin ligase that regulates the ubiquitination and degradation of IRF proteins, positioning it as a critical contributor to autoimmune disease pathogenesis.

Under physiological conditions, TRIM21 fulfills a dual regulatory function in the type I interferon pathway. Upon viral infection, it activates IFN-I signaling by tightly orchestrating the stability and activity of IRF3, IRF5, and IRF7. Once the infection is resolved, TRIM21 shifts to a negative feedback role, suppressing IFN-I production and limiting its own expression to restore immune homeostasis (80, 111). However, when factors such as autoantibodies impair the ubiquitination function of TRIM21, type I interferon signaling may become pathologically overactivated.

The following studies support this view.

In women who test positive for anti-TRIM21 autoantibodies, those exhibiting clinical signs of SLE or SS show significantly higher serum IFN-I levels (112). Anti-TRIM21 antibodies isolated from patients with SS sterically hinder the interaction between TRIM21 and E2 ubiquitin-conjugating enzymes, thereby blocking TRIM21’s RING domain–dependent self-ubiquitination (113). Anti-TRIM21 antibodies targeting the coiled-coil domain are believed to disrupt salivary gland function through E3 ligase–dependent mechanisms and are associated with increased IgG deposition in glandular tissues (114). Peripheral blood mononuclear cells (PBMCs) isolated from anti-TRIM21 positive SLE patients exhibit increased expression of IFN-I stimulated genes.

Collectively, these findings suggest that the presence of anti-TRIM21 autoantibodies impairs TRIM21’s ubiquitination function, predisposing patients to aberrant activation of the IFN-I pathway.

### 4.2 The impact of TRIM21-induced imbalance in the type I interferon signaling ubiquitination on disease

Persistent activation of IFN-I signaling represents a key pathological basis of multiple autoimmune diseases, primarily driven by aberrant activation of IRFs, particularly IRF3, IRF5, and IRF7. As a key E3 ubiquitin ligase, TRIM21 regulates the stability and activity of IRFs through ubiquitination. The following sections will examine the pathogenic roles of IRF3, IRF5, and IRF7 in different diseases, aiming to reveal potential links between TRIM21 dysregulation and tissue-specific inflammation.

In SLE and lupus nephritis (LN), IRF3 and IRF7 serve as central drivers of inflammation (115, 116). IRF3 is markedly activated in both renal tissues and peripheral blood, promoting high-level IFN-I expression. Its aberrant phosphorylation is closely associated with localized immune responses (117, 118). Sustained activation of the STING–IRF3 pathway not only drives IFN-I expression but also induces apoptosis, exacerbating tubular injury (119). Intervention studies have shown that inhibiting IRF3/7-mediated downstream gene expression significantly attenuates IFN-I–related inflammation, thereby alleviating autoimmune pathology in SLE (120).

In inflammatory bowel disease (IBD), IRF3 and IRF7 are upregulated in the intestinal mucosa, promoting localized inflammation (121). IRF5, as a key regulator of inflammation, exhibits increased activity closely associated with the development of colitis (122, 123). Inhibition of IRF5 alleviates colonic inflammation by blocking M1 macrophage polarization and dendritic cell (DC) activation (124–127).

In RA,IRF5 is markedly upregulated in synovial fluid and synovial tissues of patients. Its polymorphisms have been shown to correlate strongly with IFN-I pathway activation and the progression of inflammatory arthritis (128). In psoriasis, IRF3 and IRF7 are similarly overexpressed in lesional skin, driving IFN-α production and exacerbating local inflammation (129, 130). The regulatory role of IRF5 appears more complex; its deficiency may aggravate cutaneous inflammation by amplifying the Th17 response (131). Collectively, these findings highlight IRFs as critical regulatory nodes in the orchestration of tissue-specific inflammation. As a central transcription factor in IFN-I signaling, IRF7 is broadly activated in diseases such as SLE, psoriasis, and atopic dermatitis, enhancing local IFN responses and driving chronic inflammation.

Studies have demonstrated that TRIM21 regulates the degradation or activation of IRF3, IRF5, and IRF7 through various types of ubiquitin linkages, including K48, K63, and K27 chains. However, the specific ubiquitination patterns employed by TRIM21 in different tissues and its responsiveness to local immune microenvironments remain unclear. If TRIM21 function is impaired due to interference by anti-Ro52 antibodies, its regulatory control over IRFs may become imbalanced. This could result in sustained IRF activation and excessive production of interferon-stimulated genes (ISGs), pro-inflammatory cytokines, and chemokines by immune cells such as pDCs, macrophages, and synovial fibroblasts, thereby driving tissue-specific chronic inflammation.

Clinical observations also support a correlation between TRIM21 dysfunction and more severe phenotypes of autoimmune diseases. Patients with SS who are positive for anti-TRIM21 antibodies more frequently exhibit glandular dysfunction, parotid gland enlargement, systemic symptoms, and multiple immune abnormalities (132). High titers of anti-Ro52 antibodies are associated with severe salivary gland dysfunction, positive salivary gland biopsy, parotid gland enlargement, anemia, leukopenia, and rheumatoid factor positivity (133), and may also influence the patterns of organ involvement, particularly in the liver and muscle (134).

In summary, as outlined in **Table 1**. dysregulation of TRIM21-mediated ubiquitination may disrupt IRF homeostasis, trigger tissue-specific overactivation of type I interferon responses, and serve as a critical mechanistic link between IRF dysregulation and autoimmune pathogenesis.

**Table 1:** Expression pattern and role of IRF in autoimmune diseases.

Transcription factor	Associated diseases	Expression pattern	Functional role
IRF3	SLE/LN	Upregulated in pDCs, hyperphosphorylated in renal macrophages	Promotes IFN-α production, induces apoptosis and tubular injury
	IBD	Elevated mRNA levels in intestinal mucosa	Triggers inflammatory response in intestinal epithelium
	Psoriasis	Upregulated in skin	Enhances systemic and local IFN response
IRF5	RA	Upregulated in synovial tissue and fluid; correlated with IFN signature	Promotes M1 macrophage polarization and synovial inflammation
	IBD	Elevated in colonic mucosa; inhibition alleviates colitis	Activates inflammatory macrophages and DCs
	Psoriasis	IRF5 deficiency increases Th17 response and inflammation	May regulate Th17-mediated skin inflammation
IRF7	SLE/LN	Highly expressed in blood and kidneys; correlates with IFN levels	Master regulator of IFN-I production; essential for autoantibody generation
	Psoriasis	Overexpressed in lesional skin; drives IFN-α expression	Amplifies local inflammation
	Atopic Dermatitis	Highly expressed in lesions; identified as a pathogenic gene	Enhances IFN-I response and inflammation

*IRF*, Interferon regulatory factor; *SLE*, systemic lupus erythematosus; *LN*, lupus nephritis; *pDCs*, plasmacytoid dendritic cells; *IFN*, interferon; *IBD*, inflammatory bowel disease; *RA*, rheumatoid arthritis.

### 4.3 Clinical relevance of anti-Ro52/TRIM21 antibodies in systemic autoimmune diseases

A large number of clinical studies have demonstrated that anti-Ro52/TRIM21 antibodies are frequently detected across various systemic autoimmune diseases (9). A multicenter follow-up study examined 155 individuals who tested positive for isolated anti-Ro52 antibodies. It found that approximately 73% of these individuals were eventually diagnosed with autoimmune diseases. The most common diagnoses included SS (45–60%), SLE(35%), neonatal lupus erythematosus (>90%), and subacute cutaneous lupus erythematosus (60–100%) (109, 135–137).

In patients with SLE, anti-TRIM21/Ro52 antibodies are detected in 40–50% of cases, commonly co-occurring with anti-Ro60 and anti-La antibodies (9). Clinical studies have demonstrated that levels of anti-Ro52 antibodies are positively correlated with disease activity in patients with SLE (138). Patients who are positive for anti-Ro52 antibodies are more likely to exhibit cutaneous erythema and photosensitivity, and immunohistochemical analyses have revealed markedly increased TRIM21 expression in lesional keratinocytes (139). Anti-Ro52 antibodies are closely associated with neonatal lupus erythematosus and congenital heart block during pregnancy. Clinically, they are considered a high-risk marker for fetal cardiac involvement (140).

Anti-Ro52 antibodies represent one of the most frequently identified autoantibodies in systemic sclerosis, with a prevalence of approximately 20% (141). Epidemiological data indicate that their presence is linked to a higher risk of pulmonary arterial hypertension, interstitial lung disease, and increased mortality (141–143).

In primary Sjögren’s syndrome (pSS), anti-Ro52 antibodies are detected in up to 60% of patients and are significantly associated with disease activity, salivary gland dysfunction, vasculitis, and interstitial lung disease (ILD). High-titer anti-Ro52 antibodies are often indicative of more severe systemic involvement, including clinical features such as anemia, leukopenia, and parotid gland enlargement (133). Histological studies have revealed that TRIM21 is highly expressed in the ductal epithelium of salivary glands in pSS, with its expression closely correlated with the extent of local inflammation (9, 141) In animal models, immunization with Ro52 protein induces inflammation and functional impairment of the salivary and lacrimal glands, while passive transfer of anti-TRIM21 antibodies into healthy mice similarly reproduces glandular dysfunction and localized antibody deposition (144).

In addition, individuals who are Ro52-positive often present with more complex and severe clinical features in diseases such as Sjögren’’s syndrome, systemic sclerosis, and interstitial lung disease (ILD) (133, 141, 145, 146). However, whether anti-Ro52 antibodies have independent diagnostic value remains controversial (147).

The functional mechanisms of TRIM21 revealed in this study may help clarify this clinical controversy. TRIM21 acts as a key regulatory hub at the intersection of innate and adaptive immunity. Its dysfunction indicates a state of systemic immune dysregulation. Such dysregulation may underlie the pathogenesis of multiple autoimmune diseases, which may partly explain the broad sensitivity of Ro52 antibodies across various clinical contexts. However, the specific clinical phenotypes are influenced by individual genetic predisposition, environmental exposures, and the co-expression of other autoantibodies (e.g., anti-SSA/Ro60, anti-SSB/La, anti-RNP, anti-Scl-70). Therefore, Ro52 positivity is best interpreted as a biomarker of systemic immune imbalance, rather than a specific diagnostic marker for any single autoimmune disease.

Importantly, *in vitro* analyses have shown that anti-Ro52 antibodies are not produced at random; rather, they specifically target the essential functional domains of TRIM21, such as the RING, B-box, and coiled-coil (CC) regions, and can alter its E3 ubiquitin ligase activity by binding to these sites (113, 114, 148).

For example, antibodies that recognize the CC domain are strongly associated with salivary gland dysfunction and elevated IgG deposition (114). In contrast, antibodies targeting the RING domain have been shown to markedly suppress the E3 ubiquitin ligase activity of TRIM21 *in vitro* (113).

A more precise understanding of how autoantibodies interact with specific TRIM21 functional domains, and the functional outcomes of these interactions, could enhance the diagnostic specificity and prognostic relevance of anti-Ro52 antibodies in autoimmune diseases. Such insights may also facilitate the development of TRIM21 domain-based precision diagnostic strategies.

## 5 Discussion and conclusions

This study proposes that TRIM21 can either activate or inhibit the IFN-I pathway in different immune contexts through its diverse ubiquitination mechanisms, thereby dynamically maintaining immune system homeostasis. However, the production of anti-Ro52/TRIM21 autoantibodies may interfere with TRIM21’s ubiquitination function and disrupt its regulation of IFN-I signaling. Ultimately, this disruption can lead to abnormal activation or loss of control of the IFN-I pathway, inducing tissue inflammation and systemic autoimmune responses.

In addition to pathway-level regulation, TRIM21 exhibits remarkable cell-type specificity in its functions across distinct immune contexts. Understanding these differences is crucial for elucidating its role in immune homeostasis and autoimmune disease development.

In pDCs, TRIM21 contributes to the negative regulation of innate immune signaling. pDCs represent the primary source of type I interferons *in vivo*, and their hyperactivity is closely associated with systemic autoimmune diseases (149). Studies have shown that TRIM21 is constitutively expressed at high levels in myeloid and dendritic cell populations and can be further induced by interferon stimulation. By mediating the ubiquitination and degradation of the cytosolic DNA sensor DDX41, TRIM21 restricts IFN-I production in pDCs, thereby helping to maintain the balance between antiviral responses and immune homeostasis (105, 150).

In B cells, TRIM21 plays a regulatory role by limiting excessive humoral immune activation. It promotes the ubiquitination and degradation of the transcription factor IRF5, thereby preventing its accumulation within B cells. This process suppresses abnormal B cell activation and differentiation. In contrast, loss or dysfunction of TRIM21 drives aberrant differentiation toward a plasma cell–like phenotype, leading to increased production of antibodies and autoantibodies (84).

In macrophages, TRIM21 exerts a more complex bidirectional regulatory role. Its high expression cooperates with the proteasome to promote degradation of metabolic regulatory complexes, driving M1 polarization and enhancing the release of pro-inflammatory cytokines, thereby amplifying inflammatory responses (150). However, TRIM21-mediated ubiquitination facilitates autophagy and reactive oxygen species (ROS) clearance, contributing to anti-inflammatory effects (151).

Overall, the functions of TRIM21 across immune cell types depend on specific signaling pathways and metabolic states. It can limit excessive innate immune activation, but under certain conditions, it may also amplify inflammatory signaling. This cell type–specific regulation forms a critical basis for immune homeostasis and the development of autoimmune pathology.

In recent years, TRIM21 has emerged as a promising therapeutic target for autoimmune diseases due to its unique E3 ubiquitin ligase activity and bidirectional regulation of the IFN-I pathway. Targeted protein degradation platforms such as TrimTAC1, which leverage TRIM21’s ubiquitin ligase activity, have shown early therapeutic promise in preclinical models (152). Unlike clinically used JAK inhibitors that act by suppressing downstream signaling, TRIM21 targets an upstream component of the type I interferon (IFN-I) pathway. In the future, such strategies may complement existing therapies to more precisely address interferon signaling imbalances at their source.
