# Correction: TRIM21 regulation of IRF-mediated type I interferon signaling in systemic autoimmune diseases

**DOI:** 10.3389/fimmu.2026.1804355

**Published:** 2026-02-13

**Authors:** Kailai Panlu, Yihan Wang, Xinchao Zhu, Zhiting Lin, Ting Fang, Xinyi Yao, Zhimin Xie, Xinchang Wang

**Affiliations:** 1 The Second School of Clinical Medicine, Zhejiang Chinese Medical University, Hangzhou, China; 2 Department of Rheumatology, The Second Affiliated Hospital of Zhejiang Chinese Medical University, Hangzhou, China

**Keywords:** TRIM21, type I interferon, IRF family, autoimmune diseases, ubiquitination

An incorrect **Funding** statement was provided. The correct **Funding** statement reads:

“The author(s) declare that financial support was received for the research, and/or publication of this article. This research was supported by the National Key Research and Development Program of China (Grant No. 2024YFC3506100), the National Natural Science Foundation of China (Grant No. 82374402), the National Administration of Traditional Chinese Medicine and the Zhejiang Administration of Traditional Chinese Medicine (Grant No. GZY-ZJ-KJ-23016).”

The original version of this article has been updated.

